# Computational social science is growing up: why puberty consists of embracing measurement validation, theory development, and open science practices

**DOI:** 10.1140/epjds/s13688-023-00434-1

**Published:** 2023-12-12

**Authors:** Timon Elmer

**Affiliations:** Department of Psychology, Applied Social and Health Psychology, University of Zurich, Binzmühlestrasse 14/14, 8050 Zurich, Switzerland

**Keywords:** Computational social science, Passive-measurement, Digital trace data, Validity, Open science practices, Meta science

## Abstract

Puberty is a phase in which individuals often test the boundaries of themselves and surrounding others and further define their identity – and thus their uniqueness compared to other individuals. Similarly, as Computational Social Science (CSS) grows up, it must strike a balance between its own practices and those of neighboring disciplines to achieve scientific rigor and refine its identity. However, there are certain areas within CSS that are reluctant to adopt rigorous scientific practices from other fields, which can be observed through an overreliance on passively collected data (e.g., through digital traces, wearables) without questioning the validity of such data. This paper argues that CSS should embrace the potential of combining both passive and active measurement practices to capitalize on the strengths of each approach, including objectivity and psychological quality. Additionally, the paper suggests that CSS would benefit from integrating practices and knowledge from other established disciplines, such as measurement validation, theoretical embedding, and open science practices. Based on this argument, the paper provides ten recommendations for CSS to mature as an interdisciplinary field of research.

## Introduction

“Addressing these issues around developing useful constructs from digital trace data is the necessary spadework for the social sciences of the 21st century” (Lazer [55], p. 4)

The field of Computational Social Science (CSS) is a young one. It has proliferated in popularity, as CSS can address critical new societal phenomena – in particular, online social behavior, such as the spread of misinformation (e.g., Grinberg et al. [34]). The social, technical, and computational challenges in studying these phenomena have attracted scholars from various disciplines, such as sociology, psychology, political science, business, computer science, engineering, and physics. The interdisciplinary nature of CSS research offers many advantages; however, it also brings forward a dilemma: On the one hand, CSS research needs to be distinguishable from other disciplines – as a legitimization for its existence – while, on the other hand, CSS research needs to integrate the newly gained knowledge into existing social-science disciplines (such as sociology, psychology, or political science). As CSS is going through puberty – having established itself as an emerging field of research – it needs to advance its scientific practices to mature as a legitimate, unique, and rigorous field of research.

While there remains a debate about how CSS is defined and how it is distinguishable from neighboring disciplines (Cioffi-Revilla [14]), there seems to be a consensus that at the core of CSS is the type or size of data that is analyzed, as reflected in these three prominent descriptions:

“[…] a computational social science is emerging that leverages the capacity to collect and analyze data with an unprecedented breadth and depth and scale.” (Lazer et al. [56])

“Computational social science is an interdisciplinary field that advances theories of human behavior by applying computational techniques to large datasets from social media sites, the Internet, or other digitized archives such as administrative records.” (Edelmann et al. [20])

“[CSS is referring] to the emerging intersection of the social and computational sciences, an intersection that includes analysis of web-scale observational data, virtual lab-style experiments, and computational modeling.” (Watts [95]; p.6)

From these descriptions, it becomes evident that CSS researchers primarily analyze *passively collected data*^1^ that are generated as digital traces from individuals’ behaviors. Passive data collections do not require active input from the participants besides an initial agreement to install a tool or to use an application. Passive measurement data can be obtained with strategies such as passive sensing (e.g., Bluetooth sensors; Oloritun et al. [70]) or online trace data (e.g., web activity data; Nguyen et al. [68]). These data collection strategies stand in contrast to active data collection strategies, where participants actively contribute to the data collection, for example, by responding to a survey. Passive measurement data in online and offline settings constitutes one of the cornerstones of CSS – in contrast, for instance, to psychological research, which predominantly uses survey data in offline settings (Rafaeli et al. [77]).

However, in CSS, little discussion exists on the advantages and disadvantages of passive measurement data compared to more established types of data (e.g., survey data). Thus, in Sect. 2 of this article, I will outline some of the major advantages and challenges of passive-measurement data for social science research. On the one hand, I exemplify these advantages and challenges based on the content of Silke Adam’s keynote talk held at the 7th International Conference on Computational Social Science 2021 (IC2S2),^2^ in which she introduced the new “WebTrack” tool to track participant’s online news consumption (Adam [1]). In her talk, Adam [1] promotes a combination of passive-measurement methods and survey methods to overcome reliability issues in data collections and to generate new insights into individual-level outcomes. On the other hand, Adam’s [1] talk and her discussion of passive-measurement data serve as a starting point for a broader discussion in Sect. 2 on the advantages and challenges of using various types of passive-measurement data in CSS.

While earlier CSS research focused mainly on analyzing online-trace data, CSS is beginning to understand the value of combining data sources (e.g., see Stier et al. [86]). Along these lines, Sect. 3 will discuss the advantages of combining data sources and the potential of multi-method studies.^3^ Building on the knowledge from Sect. 2 and 3, in Sect. 4, I discuss how scientific practice within CSS can move forward. I argue that CSS needs to leverage decades of research on measurement, development and testing of theory, and production of reproducible research through open science practices. In embracing these practices, CSS will mature as a scientific field of research.

## Advantages and challenges of passive-measurement data

Before discussing the advantages and challenges of passive-measurement data, I describe the “WebTrack” data collection tool. It will be used as an example application of a passive-measurement data collection to illustrate the potential and challenges of passive-measurement data. At the same time, I will discuss examples of other types of passive-measurement data that are frequently used in CSS.

### WebTrack

The WebTrack tool is a powerful tool for studying individuals’ online information behavior. The WebTrack software functions as a screen-scraping tool that enables the tracking of online information behavior across diverse platforms, allowing extraction of the actual content a user encounters (Adam [1]; Aigenseer et al. [3]). This software employs HTML screenshots to save the content of visited URLs in real-time, effectively bypassing measurement biases often associated with retrospective tracking methods (Aigenseer et al. [3]; Mangold et al. [60]). Unlike retrospective tools that solely record the visited URL, WebTrack captures both website content and the rapidly changing elements within, such as those found on news websites (Mangold et al. [60]). Consequently, the tool offers a comprehensive depiction of participants’ website interactions. The precision of data recording extends to seconds, enabling not only the documentation of website content but also the tracking of browsing history at a granular level. Managed by the Leibniz Institute for the Social Sciences (GESIS), the WebTrack software is designed to be open source (Mangold et al. [60]).

The WebTrack tool fills an important gap in political communication research (Theocharis and Jungherr [88]) by providing a tracking tool for scientists to study individuals’ “media diets” (i.e., which information channels are chosen by users and which content they are exposed to). Tracking, in this context, refers to “every procedure intentionally applied to trace the usage of digital media aiming to analyze the collected data for research purposes” (Wieland and in der Au [97]; p. 134) and can be seen as a sub-category of passive-measurement methods.

Adam [1] argues that it is relevant to study what individuals are exposed to on the internet and how they behave in an online environment because how information is used on the internet can influence a range of individual and societal outcomes, such as health behaviors, political knowledge, or the spread of misinformation.

Although there are numerous commercial (e.g., Wakoopa, RealityMine) or academic software (e.g., Web Historian) that aid in collecting information of visited websites, the WebTrack tool has a unique combination of features, such as a high level of tracking depth, data quality, ethical standards, and being open-source and non-commercial, that is especially well suited for academic research (Adam [1]).

### Advantages

The following advantages apply to most types of passive-measurement data. They will be exemplified using the WebTrack tool and other passive-measurement methods.

#### Automatic

Once a participant agrees to activate a passive-measurement method, it records data automatically. Compared to survey data, no input is needed by the participant to collect data, leading to a low participation burden. The WebTrack tool, as an example, is installed once as a web browser add-on by the user and then automatically tracks the content of the user’s web browsing without requiring further input from the participant.^4^ Similarly, the application of wearable sensors (such as smartwatches) does not need direct input from the participant to collect data. With regards to wearable sensors, the participant additionally needs to think of wearing the device and monitor the battery level of the device.

Because of the automatized data-collection process, some forms of passive-measurement methods lend themselves well to collect data of large sample sizes in a longitudinal manner to study changes in behavior or attitudes over time. For example, Kramer et al. [52] and Matz et al. [61] conducted experiments on over 0.6 and 3.5 million Facebook users to examine the effects of personalized communication. By passively measuring participant’s behavior on Facebook, these researchers were able to collect data on a large set of individuals. In such large online experiments, however, it remains discussed how ethical standards can be adhered to (e.g., see the discussion by Flick [29]; Jouhki et al. [45]).

Although passive-measurement methods may be less burdensome to participants concerning the time they need to spend on the data collection (compared to repeatedly filling out a survey), there may be different types of burdens that participants need to take on. For example, participants need to be instructed on how to install and use a particular software (e.g., WebTrack) or how to properly wear a device (e.g., a heart rate monitoring device). Hence, for passive-measurement data it may be advisable to instruct participants on how to apply a particular software or wearable device to obtain sufficient data quality and comparable data.

#### Non-subjectivity

A major upside of passive-measurement data is that it is not biased by each participant’s subjective view. For example, the WebTrack tool records online behavior in the same way, independent of the characteristics of the participant sitting in front of the computer. Survey data often suffer greatly from subjectivity biases (e.g., memory bias, social desirability bias; Furnham [31]; Krumpal [53]; Mingay and Greenwell [65]), making between-person comparisons less justifiable. On the other hand, passive-measurement data is less prone to be influenced by participants’ characteristics or subjective perceptions.

#### Granularity

Passive-measurement data often has a high level of granularity. This granularity can concern, for example, the temporal scale (i.e., that participants’ behavior is tracked on the level of each second) or the content scale (i.e., the depth of information captured in a particular moment). The WebTrack tool has a high temporal and content granularity as it records browsing behavior on the level of each second, and it captures the content to which individuals are exposed on the internet (i.e., text, pictures, videos). As another example of a passive-measurement method, GPS location can have a high temporal and spatial granularity.

#### Always-on

Salganik [80] further argues that some passive data-collection methods are advantageous because they are “always-on”. By this, he refers to the ability of some data collection methods to record data constantly. This is, for example, beneficial when aiming to study unexpected events (e.g., collective emotions on twitter during the first COVID-19 outbreak or after terrorist attacks; Metzler et al. [64]; Steensen [84]) and to provide real-time information (e.g., during mass social events; Blanke et al. [7]; Salganik [80]). The WebTrack tool could therefore, for example, be used to study how participants inform themselves about a major news event on different news platforms (e.g., a terrorist attack).

### Challenges

The application of passive-measurement methods comes with some challenges. First, I will discuss privacy concerns. Thereafter, I will focus on challenges regarding measurement quality criteria – objectivity, reliability, and validity, as they are the most pressing ones with regards to scientific progress (for further challenges see e.g., Salganik [80]). In discussing these challenges, I will also derive some recommendations for the future of CSS.

#### Privacy

The utilization of passive-measurement data in the realms of CSS raises a multitude of privacy concerns. Since passive data collection often occurs without participants’ active engagement or direct consent at every step, concerns about privacy and informed consent become pronounced (e.g., see Flick [29]). The collection of granular digital traces from individuals’ (online) behaviors may inadvertently reveal sensitive personal information, potentially compromising their anonymity and confidentiality of oneself and others (e.g., when an Instagram feed is scraped using the WebTrack software). These challenges can be addressed by adhering to ethical standards, clear and comprehensive informed consent processes, anonymization, and by giving participants direct control over their data. For example, participants whose data are collected with the WebTrack tool, have the possibility to stop the data recording directly through the WebTrack browser extension (Adam [1]).

Another unique privacy challenge with the technical nature of most passive-measurement methods is that particular segments of the population may not participate in the data collection due to concerns about the software itself (e.g., that it transmits viruses or collects data that the participants have not agreed with). An example elucidating this challenge is provided by Gil-López et al. [32], who observed that participants who were male, young, highly educated, and politically inclined were less likely to withdraw from a web tracking study employing the WebTrack tool.

To address these privacy challenges, researchers must prioritize ethical principles and transparency in their data collection processes. *Recommendation #1:* Establish clear and comprehensive informed consent procedures, informing participants about specific types of (passive-measurement) data being gathered, the potential privacy risks involved, and how to reduce those risks.

#### Objectivity

Objectivity describes how independent a given measurement method is from researcher’s subjectivity, such as the researcher’s beliefs, feelings, and experiences (Adams [2]). While the potential biases induced by researcher’s subjectivity is generally a challenge in social sciences (Pandey [73]), the application of passive-measurement methods has a few unique challenges. Specifically, the use of electronic recording devices comes with many degrees of freedom in the choice of parameters. For example, on what level of temporal granularity are the data analyzed (Harari et al. [38])? Or based on which criteria are observations excluded? The need for a transparent discussion of data objectivity applies to passive-measurement in general as well as to the WebTrack tool specifically. As with any measurement method, the WebTrack tool is subject to potential biases that could impact the data collected. Researchers using the tool need to establish clear criteria for which observations to include or exclude in their analysis.

Transparency about factors in which researchers took decisions in a subjective, random, or theory-derived way helps in judging the objectivity criteria of the research process. One way to provide more transparency is to follow open science practices such as data and code sharing, which allow making research more reproducible, and thus less prone to researcher’s biases (for more details on open science practices, see Sect. 4). Another way constitutes a reflection and reporting of key (seemingly subjective) decisions during the research process and to provide robustness analyses for alternative decision paths. Robustness analyses (also known as sensitivity analyses) are additional analyses provided under alternative data processing or modeling conditions (Chattoe et al. [13]; Weisberg [96]). *Recommendation #2:* Establish transparency about decisions taken during the research process that might affect the objectivity of a finding and provide analyses that support the robustness of the reported findings.

#### Validity and reliability

Validity and reliability are closely intertwined, as they concern how well a measurement represents the construct that is intended to measure. Validity describes to what degree a measurement method measures the target construct (Borsboom et al. [8]; Olteanu et al. [71]). Different types of validity, such as construct and criterion validity, can be assessed – providing a broad view on what a measurement is measuring. For example, construct and criterion validity, can be assessed by means of correlating the obtained measure with other validated measurement methods of the same construct (construct validity) or theoretically related phenomena (concept validity). Reliability describes how accurately a given measurement method measures the target construct (Adams [2]; Drost [19]). Alike validity, there are different types of reliability that can be assessed. For example, the test-retest reliability describes the degree to which repeated measurements (given similar conditions) are consistent.

In psychology, for example, reliability and validity quality criteria are often closely evaluated before a measurement method is applied and used in a scientific study. For instance, the measurement methods for measuring intelligence (IQ) are validated by showing that the obtained IQ measure correlates with related real-world outcomes such as school performance (criterion validity; Gygi et al. [36]). Within psychology, it is a scientific standard to report on the reliability of a measurement (e.g., with an internal consistency coefficient; Cronbach [15]) and a reference to a validation study of a given measure.

Within CSS studies, reliability and validity should receive more attention in too, as they are rarely evaluated and reported. More research needs to be devoted to questions such as: How can we be sure that what we are measuring is actually what we are after (validity), and if so, how accurate is this (reliability)?

For passive-measurement data, it is sometimes difficult to assess its validity and reliability. Consider the WebTrack tool: Although the web content is measured “directly”, we do not know whether these data adequately represent a person’s “media diet” (the construct to be measured). Maybe the person primarily uses the smartphone (on which WebTrack is not necessarily installed) or print newspapers to obtain political information, and therefore the WebTrack tool potentially provides an imprecise (i.e., unreliable) picture of a person’s media diet.^5^ In her talk, Adam [1], highlights how the combination of passive-measurement data together with survey data on news consumption may help to obtain a more reliable picture of a person’s media diet (also see Sect. 3).

If we derive measures from the WebTrack data, for example, to classify web pages into those with political and non-political contents, it is difficult to judge whether a classifier validly categorizes this content. Adam [1], for example, describes how human-coded ratings about the absence or presence of political content were used to train supervised machine learning algorithms on the WebTrack data to detect which web pages contain political content. This is a laudable way of assessing the criterion validity of machine learning algorithms.

Another type of validity was examined by Gil-López et al. [32]. They investigated the external validity (i.e., the generalizability) of the WebTrack tool and found that certain participant characteristics predicted participation dropout. Hence, the generalizability of findings may be limited to certain population groups.

While the research around the WebTrack tool entails many examinations of measurement quality criteria, in CSS research in general, there may still be a lack of systematic evaluations of the validity and reliability of measures. When claiming that one measure *X* is statistically associated with an outcome measure *Y*, we must be able to convincingly argue that the measurements of *X* and *Y* approximate the construct of interest by examining validity and reliability. *Recommendation #3*: Conduct validity and reliability studies of frequently used measures in CSS.

The call for more attention to better measurement evaluations – including reliability and validity – is not new (e.g., Jacobs and Wallach [42]; Lazer [55]; Ruths and Pfeffer [79]; Tufekci [91]). Fortunately, efforts to test the reliability and validity of measurements have been appearing within CSS (e.g., Pellert et al. [74]).

For example, Radio Frequency Identification (RFID) badges have been widely applied to measure face-to-face social interactions (e.g., Cattuto et al. [12]; Elmer and Stadtfeld [25]; Smieszek et al. [83]). But only recently have they been validated for the measurement of face-to-face social interactions in social gatherings by comparing the RFID data with hand-coded video data (construct validity) and self-reports of social interactions (criterion validity; Elmer et al. [24]). Further validation studies, for example, on the application of RFID badges in a variety of social settings, are needed.

When measures are used in empirical articles, the reader should be informed about whether validity or reliability studies exist of the particular measure and how well the measure measures the underlying construct. *Recommendation #4*: Discuss reliability and validity indicators of in measurements sections of CSS articles.

## Advantages of combining data sources

I will illustrate the advantages of combining data sources using the WebTrack-research described by Adam [1] in her IC2S2 keynote talk, as most of her talk consisted of a similar goal. In later parts of this section, I will extend these arguments also to other fields of CSS research.

Adam [1] did not use the WebTrack tool by itself but combined it with survey data from before- and after the WebTrack assessment phase. In combining passive-measurement methods with survey methods, Adam [1] provides two arguments for why this is beneficial. First, she argued that connecting individual media diets to survey data allows researchers to predict individual-level outcomes – such as changes in political trust during the COVID-19 pandemic (de León et al. [17]). The strength of survey data is to capture valuable *subjective psychological data* about individual’s internal thoughts and feelings (e.g., political trust), which – in combination with passive-measurement data – can lead to a better understanding of social and individual phenomena. In other words, to overcome the challenge that we often do not know what participants think or feel when behaving in a particular way, we need to consider active input from the participant in the form of survey or text data to assess these psychological states.

Second, Adam [1] argues that we can combine passive-measurement data with survey-based offline exposure data (e.g., which print newspapers an individual regularly read) to obtain a complete picture of an individual’s media diet (*augmentation*), which improves the reliability of data. In other words, media diets are more accurately assessed by combining data sources. This way, online and offline behavior need not be treated as separate empirical entities. While neither form of data may be completely reliable or valid due to its unique advantages and disadvantages (see Sect. 2), their combination (and differentiation) may provide a more nuanced picture of an individual’s behavior. In smartphone-usage research, for instance, researchers are discussing which type of information (active vs. passive reports of smartphone usage) may be applicable for what purposes (e.g., Araujo et al. [6]; Ellis et al. [22]).

There are additional reasons for combining active and passive-measurement methods. Third, a multi-methods approach can be beneficial to validate passively-collected data with survey-based measures. At the same time, active measurements should not be treated as the ground truth, as survey methods are just another tool in a toolkit where there is no one “true” measure of many social scientific concepts.

Stier et al. [86] argue about survey and digital trace data that “there are relatively few studies that combine these two data types” (p. 503). Yet, within recent years, more and more (CSS) studies use a combination of passive-measurement data and survey data to leverage the strengths of each type of measurement method (e.g., Al Baghal et al. [4]; Elmer and Stadtfeld [25]; Stier et al. [85]). For example, Guess and colleagues [35] linked Twitter and Facebook account data to survey data and compared their overlap in self-reported and observed (passively sensed) political social media use. They found that some individual characteristics, such as age and partisanship, are predictive of over-self-reporting on Facebook, but not on Twitter. Recently, Langener et al. [54] published a review on the combined application of passive-measurement methods and daily-life surveys (Experience Sampling Methods; e.g., Csikszentmihalyi and Larson [16]; Trull and Ebner-Priemer [90]) to capture participant’s social context. While highlighting the importance of combining these data sources, they conclude that there is a strong need for better-validated measurement methods.

There seem to be three types of studies that combine survey and passive-measurement data. On the one hand, there are studies that compare survey and passive-measurement data (e.g., Araujo et al. [6]; Ellis et al. [22]; Guess et al. [35]). This type of study may be useful to examine the construct validity of a given measure or to learn more about the discrepancy between self-reports and the “actual” behavior of participants (i.e., social desirability bias). On the other hand, there are studies that use a multi-method approach in which the outcome and the predictor variable are measured with different methods: One data source comes from passive-measurement data, while another source is survey responses. For instance, studies that examine the association between (self-reported) mood and (passively-sensed) smartphone usage (e.g., Bradley and Howard [9]). As the common-method bias is reduced, multi-method studies allow for a more robust examination of associations (Jordan and Troth [44]). A third type of study generally aims to combine measurement methods in order to better classify an outcome variable. One line of research, for example, tries to classify individuals into “depressed” and “non-depressed” groups based on their data from self-reports *and* from wearables or smartphones (e.g., Moshe et al. [67]; Opoku Asare et al. [72]). The conduction of these three types of studies is important for CSS to move forward, as they examine in more detail what passive-measurement data can help to uncover what self-reports (maybe) cannot.

Yet, combining these data methods does not come without challenges (also see a special issue from 2020 on this topic in *Social Science and Computer Review*). Stier et al. [86] have discussed three key challenges when combining survey and digital trace data. First, there is the challenge of obtaining consent from participants for combining data sources. This may be a challenge in studies where participants are not completely aware of their participation (e.g., by being part of a social media experiment, Flick [29]; Kramer et al. [52]; Matz et al. [61]) or when data linkage of different sources is done post-hoc (Stier et al. [86]). Second, there are ethical and methodological issues – such as the external validity of online-behavior for understanding offline behavior (e.g., Jürgens et al. [48]). Third, the lack of conceptual and theoretical frameworks incorporating both types of data, such as the processing of data types with different temporal granularity (Langener et al. [54]; Stier et al. [86]). Despite these challenges, the combination of data sources holds great potential for the joint study of digital social behavior.

## Ways forward (into adulthood)

Being the “child” of computer science and social sciences, there are great opportunities for CSS to contribute to understanding societal phenomena – but there are also challenges to be overcome when growing up.

It has been argued that CSS as an “interdisciplinary research field struggles with establishing practices that connect it more strongly within the established social sciences, develop standards of transparency in data collection, preparation, harmonization and analysis, and surface and problematize conflicts of interest between researchers, industry, and the media” (Theocharis and Jungherr [88]; p. 7). Along these lines, I argue that we need better integration of CSS research(ers) in the established social sciences by following gold-standard scientific practices – such as those discussed in the remainder of this section. This way, CSS can mature and be recognized by “classical” social scientists as a legitimate interdisciplinary research field. These areas for important developments in CSS concern specifically (a) theoretical embedding, (b) conceptualization and measurement validation, and (c) open science practices. Each of these areas will be discussed in the remainder of this section. I close this section by discussing the importance of boundary definitions.

### Theoretical embedding

Various scholars have criticized CSS research for its limited theoretical embedding (e.g., Edelmann et al. [20]; Jungherr and Theocharis [47]; Rains [78]; Wise and Shaffer [98]). It is argued that more focus has been put on easily measurable online metrics (Jungherr [46]) or mathematical modeling (McFarland et al. [63]) than on theoretical embedding. Yet, without proper theoretical embedding, even the fanciest data collections cannot contribute to advancing knowledge – or as Borsboom et al. [8] put it: “no amount of empirical data can fill a theoretical gap” (p. 1068).

As part of every empirical CSS project, scholars should invest time in building theoretical models of *why* the focal predictor variable(s) *X* might be associated with the focal outcome variable(s) *Y* (Hedström and Ylikoski [39]; Kellen [50]).

As a positive example, de Léon and colleagues [17] discuss a mechanism underlying their hypothesis that the “consumption of alternative anti-establishment news media is related to decreases in political trust during the first wave of the COVID-19 pandemic” (p. 6). They argue that alternative news media tend to be “highly critical of public institutions and established political actors” (p. 6) and that their consumption “is linked to reduced willingness to follow government policies” (p. 6), which should also manifest in reduced trust – constituting another manifestation of the non-following of governmental policies. This way, de León et al. [17] discuss the assumed theoretical mechanism underlying their hypothesis, which they test on a combination of survey and WebTrack data.

Even when a hypothesized association (e.g., a correlation) is shown to be present in the data, the theoretical work is not done yet. A crucial last step is missing, that is, the integration into existing knowledge and an updated theoretical model (also see research cycles by Valsiner [92]; Wagenmakers et al. [94]). Speculations about mechanisms on why *X* might be associated with *Y* can inform future research investigating these specific mechanisms (e.g., through mediation analysis). *Recommendation #5*: Include an argumentation and discussion about the theoretical mechanisms of an investigated statistical relationship.

Mechanisms can be part of a larger theory – aiming to explain a particular (set of) outcome(s), and empirical evidence can be subsumed into a general theory. Consider, for example, Darwin’s theory of evolution.^6^ He carefully collected empirical evidence on the distribution of bird species across different islands. Over the course of many years, he analyzed those patterns and developed a theory that can account for the variety of outcomes in bird features. The theoretical work of systematically comparing these patterns and coming up with a general mechanism could not have been done by computational tools (e.g., a machine learning algorithm) but required a lot of biological and geographical knowledge to bring together these observations into a theory.

The overt focus of CSS on big data and computational modeling (Jungherr [46]; McFarland et al. [63]) puts the attention away from comprising existing evidence or personal observations into testable theories. Hence, for CSS to advance, more emphasis needs to be put on theory development.

For theory development, classical social scientific skills are required. Despite CSS’s focus on the analysis of large, passive-measurement data, one should not forget about the (complex) work of theory development, as it constitutes an essential step in scientific progress. Within the psychological sciences, the process of theory development has been underrepresented, leading to claims that psychology is facing a “theory crisis” (e.g., see Eronen and Bringmann [26]; Fried [30]). Let us hope that this does not happen to the field of CSS. *Recommendation #6*: Invest in the development and (empirical and conceptual) testing of theories.

### Conceptualization and measurement validation

Any kind of theory (or research question) needs to be examined with well-conceptualized and well-measured phenomena. An important – but often neglected – prerequisite for measuring a concept is that the concept is well-conceptualized, that is (at least), that the concept has a clear definition (Bringmann et al. [10]; Flake and Fried [28]). Consider the example of the concept of friendship. Although friendship networks have been widely studied with a one-item measure, existing attempts to define friendships remain somewhat vague, thereby leaving much room for interpretation – making it difficult to compare its measure on an intra- and interindividual level (Bringmann et al. [10]; Fischer [27]). Another example comes from research on online social interactions, where Hall [37] has demonstrated that online behavior that is sometimes coined as a social interaction (e.g., ‘liking’, ‘re-tweeting’) does not align theoretically and empirically with what researchers and participants see as a social interaction. Another example comes from web-tracking data on news sites and the study of political trust (e.g., de León et al. [17]): While it may be possible to measure political trust, it may not be so clear whether participants and researchers have similar concepts in mind when thinking about the word “trust”. *Recommendation #*7: Provide definitions of key concepts in the introduction section of research articles.

Only when there is a consensus among the researchers that aim to study a particular phenomenon can a valid measure be developed. In developing a survey-based measure, survey items are often derived from the definition of the studied concept (Moosbrugger and Kelava [66]). However, when using passive-measurement data, this crucial step of conceptualizing can easily be forgotten because the measurement is often pre-determined by technical possibilities. For example, the concept of a “social interaction” has been measured using a variety of sensors (RFID, Bluetooth, WiFi) that measure proximity with different levels of spatial granularity (e.g., Cattuto et al. [12]; Madan et al. [59]; Sapiezynski et al. [82]). For example, RFID sensors measure proximity at ranges up to 1.6 meters, whereas Bluetooth-sensed proximity reaches up to 10 meters and WiFi up to 45 meters (Elmer et al. [24]; Sapiezynski et al. [81]). As a result of the heterogeneity in spatial granularity, the question remains open to what extent these methods measure comparable types of social interactions. I believe that (each of these) these passively sensed measures of social interactions have their merits but that the (mis-)match between the concept and the measurement practice should be openly discussed. *Recommendation #7*: Discuss the match between the concept definition and its measurement in empirical articles.

One way to assess how well the measurement method measures the focal concept is through validation studies (also see Sect. 3 and *Recommendation #2*). In validation studies, the measurement method is often compared to a reasonable external measure (e.g., expert report; content validity) of the same construct (i.e., construct validity). An interesting comparison study in the realm of measuring social interactions with passive-measurement data would be to compare the overlap of RFID, Bluetooth, and WiFi sensors with self-reports of social interactions or video-based reports of social interaction, which are coded based on a common definition of what constitutes a social interaction.

Another type of validity that is important to CSS studies is external validity, which reflects to what extent the measure is associated with phenomena that happen outside of the study context – in other words, how generalizable the findings are (Flake and Fried [28]; Olteanu et al. [71]). When studying participants’ online behavior, it would be relevant to know, for example, how much the popularity of politicians within a twitter network (e.g., Lietz et al. [58]) corresponds to their popularity in the general population, such as measured with official voting data. Otherwise, one might run into the danger that the Twitter network is seen as an adequate representation of the real political landscape.^7^

It is, furthermore, key that we are transparent in what is measured and *how* this measure is interpreted to make a more general claim. Let us, for instance, consider that individual *A* has 73 million followers on Twitter (recently renamed to *X*) and individual *B* has 35 million followers. We could derive that individual *A* has more influence in the Twitter sphere than individual *B*. Would you also say that individual *A* is generally a more influential person than individual *B*? Naturally, we might be tempted to interpret this difference in the follower measure as individual *A* being twice as influential as individual *B*. Both are important in the Twitter sphere, but in different ways, and this influence in online social media networks might not reflect the offline influence that these two individuals have.

As this example illustrates, the step from a measure to its interpretation is thus always straightforward. Measures generally have an *interpretation boundary*, that is, measures can only be interpreted with regard to what they are measuring and not beyond. In our example with individuals *A* and *B*, we could interpret the follower count measure as a reachability measure (i.e., that individual *A* can directly reach more Twitter followers than individual *B*), but we would cross the interpretation boundary if we would claim that individual *B* is a more *influential* person than individual *A*.^8^ That is not to say that the online sphere is not worth studying, but rather that the interpretation of online-derived measures is bound to the online sphere.

### Open science practices

These theoretical and methodological challenges are closely linked to reproducibility efforts and the fostering of open science practices. Open science practices within CSS could entail “open practices, open data, open tools, and open access” (Voelkel and Freese [93], p. 1). These practices aim to make research as transparent, open, and reproducible as possible (Nosek et al. [69]). The development of measurement methods or computational measures needs to be documented and justified so that results obtained with the given measure can be reproduced by other scholars. For example, the WebTrack app is an open-source software that can be used by other scholars to reproduce the results in a different sample and setting.

The reproducibility crisis has also reached disciplines in which machine learning is prominent (Kapoor and Narayanan [49]), making it even more urgent that open science practices are promoted to allow other researchers to better understand what was exactly done in a study.

An excellent example of a CSS study (although not labeled as such) applying some open science practices is the one of Eichstaedt et al. [21]. Eichstaedt and colleagues used Twitter data to predict county-level heart disease mortality in the US. They made their data and materials openly available on the platform of the Open Science Framework (osf.io/rt6w2). This way, other researchers have the opportunity to reconstruct the analysis. Brown and Coyne [11] did this and wrote a commentary paper about their reanalysis, leading to an inspiring open discussion about important aspects of the analysis. I argue that examples like these bring the field forward. While such commentaries using openly available data are more common in psychology (also see e.g., Elmer [23]; Quoidbach et al. [75, 76]), they have remained fairly infrequent in CSS – potentially due to the lack of open science practices. This is one example where CSS can learn from neighboring disciplines (in this case, from psychological sciences) to obtain more scientific rigor and recognition.

While sharing data openly may be beneficial for scientific progress, one needs to carefully consider and assess privacy concerns when doing so. Ethical guidelines to ensure participant’s privacy and anonymity must be kept ensured even when parts of the data are publicly shared. If a company provides the data, the shared data must oblige to the (sometimes very restricted) data sharing policies of a given company that provides the data (for a discussion on such obstacles, see Lazer et al. [57]). One way to overcome these ethical and regulatory challenges is to publish *processed* data.

As a large proportion of CSS research is interested in the association between an independent variable *X* and a dependent variable *Y*, while controlling for variable(s) Z, the processing of these variables *X*, *Y*, and *Z* should be possible without giving away sensitive information.

GPS data, for example, is very sensitive as it may disclose individuals’ frequent locations (e.g., their homes). Yet, for using GPS distances as an independent variable (e.g., Depp et al. [18]) one does not require to publish the exact GPS locations of the participants. Data on, for instance, the distances traveled can be openly published without giving away sensitive information on the location of participants. Similarly, the data that is collected through the WebTrack application may contain sensitive information (e.g., usernames and specific websites that are visited). When sharing such data openly, it is important to process the data in a way that preserves the privacy of participants (e.g., by only reporting aggregated statistics of how long a person was exposed to a particular type of information on a website).

The processing of data may not be an objective and transparent process; hence, open science practices also entail making transparent how exactly the raw data was processed – ideally by sharing code. The GPS and the WebTrack example show that the preservation of participant’s anonymity and open science practices do not have to rule each other out. For a more detailed discussion on privacy, open data practices, and data processing see, for example, Joel et al. [43] and Towse et al. [89].

In some social science fields, the application of open science practices is rewarded with a “open science badge” system (e.g., a paper gets an “open data badge”, if the data is made publicly available; Grahe [33]; Kidwell et al. [51]). There have been calls to standardize open science practices within CSS, but only recently Hofman [40] and not with enough systematic reward. *Recommendation #9*: Engage in the adoption and reward of open science practices (i.e., open data, open code, open access).

### Boundary definitions

While the three above-introduced ways forward (i.e., theoretical embedding, conceptualization and measurement validation, open science practices) concern topics on the level of specific studies, the topic of boundary definitions is one that concerns the image of CSS as a research field.

Studies that use digital trace data, similar to those in CSS, are becoming increasingly common in psychology, yet they are not always classified as CSS studies (e.g., Eichstaedt et al. [21]). One possibility to explain this classification is that the term CSS does not always coincide with positive affirmation – possibly because CSS researchers rely too heavily on the size of their data. For example, Hox [41] argued that in CSS, “there is the problem of (lack of) transparency, the issue that the size of the data by itself is not a quality indicator, the meaning of veracity, and the question how well big data analytics works on smaller data” (p. 10).

So what are the reasons for these image problems of CSS? One reason may lie in the slow uptake of rigorous scientific practices concerning measurement quality criteria (i.e., objectivity, reliability, validity) and open science practices (see above). Psychology has made the mistake of not considering these aspects early enough – leading to a replication crisis (Maxwell et al. [62]) and a general distrust in psychological research findings (Anvari and Lakens [5]).

Another reason may lie in the fuzzy boundary definitions of CSS. When is a study a CSS study? As soon as it starts to use passively measured (digital) data? It may be beneficial that research fields are defined based on the types of research questions that they are trying to answer instead of the type (or size) of data that is used. The term computational may suggest to scholars of other disciplines that they are not “really” using computational tools to study questions in their field, but even the simplest regression model is a computational procedure. In the words of Theocharis and Jungherr [88]: “As in the most general reading of our [CSS] definition the use of any computational method in data handling and analysis would qualify as computational social science, one could argue that nearly any form of contemporary social science would constitute computational social science” (p. 4). This quote raises the question of what the boundary definitions of CSS are. There seems to be no clear consensus on this question, hampering the process of disseminating CSS as a legitimate research field.

Nevertheless, I argue that CSS research has the potential to stay its own legitimate and valued field of research if it manages to adapt practices and theories of “grown-up” disciplines and manages to rebrand its focus away from using big data and complex computational models to focus on the understanding and explanation of human behavior in digital social spheres, which is what the majority CSS researchers seem to conduct research on. Therefore, a strategic “rebranding” of the term CSS appears advantageous, involving a shift in emphasis from non-distinctive attributes like data volume or computational methodologies towards more distinctive attributes, notably the nuanced comprehension of human behavior within digital realms. *Recommendation* #10: Rebranding CSS as an interdisciplinary research field that aims to understand and explain human behavior in digital social spheres.

## Conclusion

“Another important barrier is that much of CSS research appears to lack connections to relevant theories, [and] deploys measures that can be questionable […]. These are all understandable symptoms of an interdisciplinary field that has not yet matured, and they can, as numerous textbooks demonstrate, also be encountered in other types of social science research […]” (Theocharis and Jungherr [88], p. 3).

Along those lines, I have argued in this article that the adaptation of existing (well-researched) practices from other disciplines holds great potential for the development of CSS as a (more) legitimate scientific field of research. I have mainly focused on the fruitfulness of combining passive- and active-measurement data while touching upon similarly relevant topics – measurement validation, theoretical embedding, and open science practices. Table 1 summarizes ten recommendations for CSS to move forward as a scientific research field. Although CSS has had a flourishing childhood and has grown rapidly in scientific and public recognition, the next phase of puberty will show how well CSS can integrate itself into the community of existing disciplines by being open to learning from them and thus mature as a research field.

**Table 1:** Overview of Recommendations

Category	Recommendation
Privacy	Establish clear and comprehensive informed consent procedures, informing participants about specific types of (passive-measurement) data being gathered, the potential privacy risks involved, and how to reduce those risks
Objectivity	Establish transparency about decisions taken during the research process that might affect the objectivity and provide analyses that support the robustness of the reported findings
Measurement	Conduct validity and reliability studies of frequently used measures in CSS
	Discuss reliability and validity indicators of in measurements sections
Theory	Include an argumentation and discussion about the theoretical mechanisms of an investigated statistical relationship.
	Invest in the development and (empirical and conceptual) testing of theories.
Conceptualization	Provide definitions of key concepts in the introduction section of research articles.
	Discuss the match between the concept definition and its measurement in empirical articles.
Open Science Practices	Engage in the adoption and reward of open science practices (i.e., open data, open code, open access).
Boundary Definitions	Rebranding CSS as an interdisciplinary research field that aims to understand and explain human behavior in digital social spheres.

## Data Availability

Data sharing is not applicable to this article as no datasets were generated or analysed during the current study.

## Footnotes

1 I am not considering CSS research that solely relies on simulation (e.g., agent-based models) without empirical data.

2 One goal of this EPJ Data Science special issue was to discuss the keynote talks of IC2S2 2021. Hence, the content of Silke Adam’s keynote talk is relatively prominent in this article.

3 Multi-method designs, including different types of data collections, are not to be confused with mixed-methods design, which entail a mix of qualitative and quantitative data collection methods (Tashakkori et al. [87]).

4 Participants see in their browsers when WebTrack is actively recording. The participants can manually pause the tracking of data.

5 One way to further assess the construct validity of WebTrack is to conduct laboratory experiments in which participants may use their device of choice (e.g., computer, smartphone) for media consumption. By comparing data from WebTrack with the participant’s own observations of media consumption and independently-rated video data of the device’s screens, one may obtain more information on the validity of WebTrack to measure media diets. While this approach may not reflect participants’ behaviors in daily life, it may represent an intermediate step to better understand the validity of passive-measurement tools such as WebTrack.

6 This example is inspired by Eronen and Bringmann [26].

7 This was the case in the example of Lietz et al. [58], where members of the “pirate party” were overrepresented in the twitter network.

8 Individual A is Kim Kardashian (a popstar) and individual B is Joe Biden (current US president).

## Abbreviations

CSS: Computational Social Science
IC2S2: International Conference on Computational Social Science
HTML: Hypertext Markup Language
URL: Uniform Resource Locator
GESIS: Leibniz-Institut für Sozialwissenschaften (ehemals Gesellschaft Sozialwissenschaftlicher Infrastruktureinrichtungen)
RFID: Radio Frequency Identification
GPS: Global Positioning System
